# Synthesis and Biological Evaluation of Novel Benzothiazole-2-thiol Derivatives as Potential Anticancer Agents

**DOI:** 10.3390/molecules17043933

**Published:** 2012-03-30

**Authors:** Xuan-Hong Shi, Zhao Wang, Yong Xia, Ting-Hong Ye, Mei Deng, You-Zhi Xu, Yu-Quan Wei, Luo-Ting Yu

**Affiliations:** State Key Laboratory of Biotherapy, West China Hospital, West China Medical School, Sichuan University, Chengdu 610041, China

**Keywords:** anticancer, synthesis, apoptosis, benzothiazole-2-thiol derivatives

## Abstract

A series of novel benzothiazole-2-thiol derivatives were synthesized and their structures determined by ^1^H-NMR, ^13^C-NMR and HRMS (ESI). The effects of all compounds on a panel of different types of human cancer cell lines were investigated. Among them, pyridinyl-2-amine linked benzothiazole-2-thiol compounds **7d**, **7e**, **7f** and **7i** exhibited potent and broad-spectrum inhibitory activities. Compound **7e** displayed the most potent anticancer activity on SKRB-3 (IC_50_ = 1.2 nM), SW620 (IC_50_ = 4.3 nM), A549 (IC_50_ = 44 nM) and HepG2 (IC_50_ = 48 nM) and was found to induce apoptosis in HepG2 cancer cells.

## 1. Introduction

A number of benzothiazole derivatives have exhibited interesting biological activities [1,2,3] and attracted continuing interest for further molecular exploration as useful anticancer agents [4,5]. Our preceding studies had found that two benzothiazole-2-thiol compounds (compounds **1** and **2**) displayed good anticancer activities and induced HepG2 cell apoptosis *in vitro* [6]. In order to develop more potent tumor growth inhibitors as novel anticancer agents, we designed and synthesized a series of novel benzothiazole-2-thiol derivatives through incorporation of heterocyclic rings (pyridine, pyrimidine and thiazole) to benzothiazole-2-thiol derivatives with the activity and safety advantages of the heterocyclic ring structures [7,8]. The effects of all the novel compounds on a panel of different types of human cancer cell lines were investigated by the MTT assay and compound **7e** was selected to examine apoptosis on HepG2 cell cells by flow cytometry. As a result, the pyridinyl-2-amine linked benzothiazole-2-thiol compounds exhibited potent anticancer activities and compound **7e** inhibited the proliferation of HepG2 cell via inducing apoptosis.

## 2. Results and Discussion

### 2.1. Chemistry

**Scheme 1:**
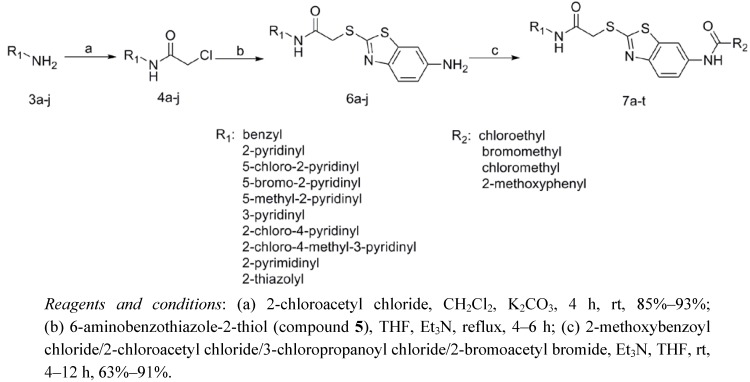
Synthetic route for **7a–t**.

Twenty novel benzothiazole-2-thiol derivatives linked with heterocyclic rings were designed and synthesized by the route shown in Scheme 1.

Commercially available amines (compounds **3a–j**) were first reacted with 2-chloroacetyl chloride in the presence of potassium carbonate as the base in dichloromethane to give crude compounds **4a–j**. The raw products **4a–j** were puriﬁed by recrystallization from ethyl acetate/petroleum ether. The compounds **6a–j** were prepared by reacting **4a–j** with 6-aminobenzothiazole-2-thiol (compound **5**) and triethylamine (TEA) as the base in tetrahydrofuran (THF) under reflux. Compounds **6a–j** respectively were thus obtained and could be used directly for the next step without further puriﬁcation. The reaction mixtures of compounds **6a–j** were further reacted with 3-chloropropyl chloride, 2-bromoacetyl bromide, 2-chloroacetyl chloride and 2-methoxybenzoyl chloride in the presence of triethylamine (TEA), respectively. The precipitates were collected by filtration and washed with water to yield the crude products (compounds **7a–t**). Each compound was puriﬁed by column chromatography on silica gel using petroleum ether/ethyl acetate as eluent. The structures of all compounds were determined by ^1^H-NMR, ^13^C-NMR and HRMS (ESI).

### 2.2. Biological Activities

The twenty novel synthesized benzothiazole-2-thiol derivatives were investigated for anticancer activity *in vitro *on cancer cell lines by the MTT assay with compounds **1** and **2** as positive controls. Compounds **1** and **2** showed good activity against human colon adenocarcinoma cell line (SW480), human cervical cancer cell line (HeLa) and human hepatocellular carcinoma cell line (HepG2) in our preceding studies. Herein, we selected firstly the three human cell lines to test these compounds and the results were presented in Table 1.

**Table 1:** The anti-proliferative activities for compounds **1**, **2** and **7a–t**.

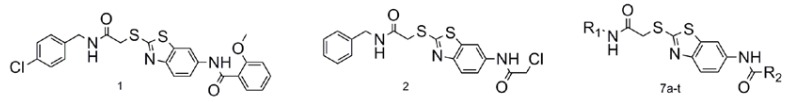					
Compd.	R_1_	R_2_	IC_50_ (µM) ^a^		
			HepG2	SW480	HeLa
**1**	4-chlorobenzyl	2-methoxyphenyl	0.7	5.6	4.0
**2**	benzyl	chloromethyl	1.0	5.2	4.6
**7a**	benzyl	chloroethyl	5.6	15.7	8.6
**7b**	benzyl	bromoethyl	6.6	14.9	48.1
**7c**	2-pyridinyl	chloromethyl	0.7	2.3	3.1
**7d**	5-chloro-2-pyridinyl	chloromethyl	0.26	0.46	0.035
**7e**	5-bromo-2-pyridinyl	chloromethyl	0.048	0.68	0.02
**7f**	5-methyl-2-pyridinyl	chloromethyl	0.091	1.0	0.03
**7g**	3-pyridinyl	chloromethyl	6.6	6.4	12.6
**7h**	2-chloro-4-pyridinyl	chloromethyl	5.0	2.9	1.9
**7i**	2-chloro-4-methyl-3-pyridinyl	chloromethyl	0.4	2.4	0.4
**7j**	2-pyrimidinyl	chloromethyl	24.3	54.8	24.3
**7k**	2-thiazolyl	chloromethyl	13.1	7.1	14.5
**7l**	2-pyridinyl	2-methoxyphenyl	>100	>100	>100
**7m**	5-chloro-2-pyridinyl	2-methoxyphenyl	>100	>100	0.6
**7n**	5-bromo-2-pyridinyl	2-methoxyphenyl	23.5	23.8	23.5
**7o**	5-methyl-2-pyridinyl	2-methoxyphenyl	45.6	31.1	0.8
**7p**	3-pyridinyl	2-methoxyphenyl	>100	>100	>100
**7q**	2-chloro-4-pyridinyl	2-methoxyphenyl	>100	>100	>100
**7r**	2-chloro-4-methyl-3-pyridinyl	2-methoxyphenyl	23.1	>100	34.6
**7s**	2-pyrimidyl	2-methoxyphenyl	27.2	>100	>100
**7t**	2-thiazolyl	2-methoxyphenyl	>100	>100	>100

**^a^** Values are means of three experiments.

These novel compounds showed great variation of IC_50_ values on the three cell lines and compounds **7d**, **7e**, **7f** and **7i** exhibited potent inhibitory activities. To further study the cytotoxic profile, the potent analogues **7d**, **7e**, **7f** and **7i** were selected for further evaluation of inhibitory activities against other eleven types of human cancer cell lines, including colon cancer cell lines HCT-116 and SW620, lung cancer cell line A549, prostate cancer cell line PC-3, pancreatic cancer cell line BxPC-3, breast cancer cell line BT474, epidermoid cancer cell line A431, ovarian cancer cell line SKOV-3, non-small cell lung cancer cell line H460, breast cancer cell line MDA-MB-468 and SKRB-3.

As shown in Table 2, compounds **7d**, **7e**, **7f** and **7i** exhibited potent and broad-spectrum anticancer activities which were much better than compounds **1** and **2**. Among them, compound **7d** showed the most potent antitumor activities against A431 (IC_50_ = 20 nM) and compound **7e** displayed the most potent anticancer activity on SKRB-3 (IC_50_ = 1.2 nM), SW620 (IC_50_ = 4.3 nM), A549 (IC_50_ = 44 nM) and HepG2 (IC_50_= 48 nM). The antitumor activities of compounds **7d** and **7e** were about 10–1,000 times greater than compounds **1** and **2** (SW620, A549, SKRB-3 and HepG2). These results suggested that pyridin-2-amine linking benzothiazole-2-thiol derivatives have potent and broad-spectrum anti-cancer activities and be worth being further investigated as candidate of anticancer agent.

**Table 2:** The anti-proliferative activities for compounds **7d**, **7e**, **7f**, **7i**, **1** and **2** against various cancer cell lines.

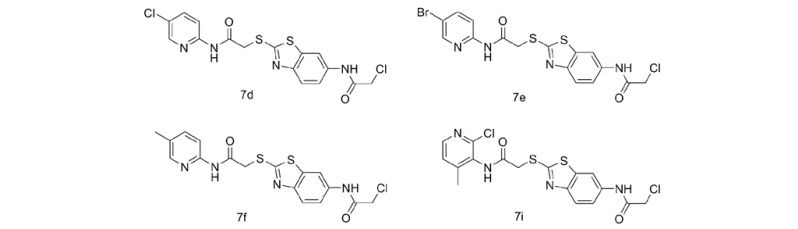											
Compd.	IC_50_(µM) ^a^										
	HCT116	BT474	SW620	H460	PC-3	BXPC-3	A431	A549	SKOV-3	MDA-MB-468	SKRB-3
**7d**	0.8	4.3	0.033	3.7	7.9	0.1	0.02	0.2	2.7	1.5	0.5
**7e**	0.3	0.6	0.0043	0.3	6.0	0.3	0.2	0.044	0.4	0.9	0.0012
**7f**	0.6	5.3	1.5	4.2	6.8	0.5	0.1	0.3	3.5	2.5	1.9
**7i**	2.2	5.2	6.5	14.6	8.6	9.3	4.8	8.3	5.8	6.8	11.3
**1**	1.6	>100	4.1	39.7	4.8	4.7	1.0	2.0	6.0	2.4	1.5
**2**	1.2	7.8	1.3	10.0	6.0	6.6	4.5	4.4	4.6	4.0	8.0

**^a^** Values are means of three experiments.

**Figure 1:**
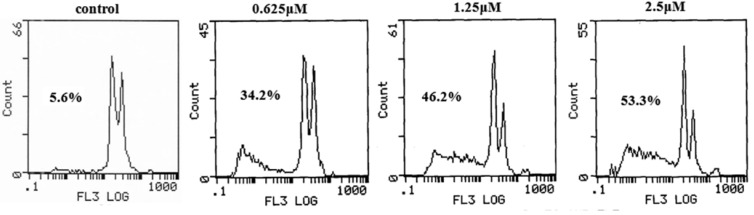
Compound **7e** concentration-dependently induced apoptosis in HepG2 cancer cells.

In order to investigate the apoptosis effects of the benzothiazole-2-thiol derivatives, we selected compound **7e** to examine apoptosis effects on HepG2 cells by flow cytometry. Flow cytometric analysis was performed to measure the apoptotic cells and the cell cycle after propidium iodide (PI) staining [9]. From Figure 1, the percentages of apoptotic cells were 34.2%, 46.2% and 53.3%, respectively, with 0.625 µM, 1.25 µM, and 2.5 µM compound **7e** treatment for 24 h. The results indicated that compound **7e** inhibited the proliferation of HepG2 cell via inducing apoptosis on a concentration-dependent manner. The exact and further biological mechanism of compound **7e** is under investigation in our laboratory.

## 3. Experimental

### 3.1. General

The human cancer cell lines were purchased from the American Type Culture Collection (ATCC, Rockville, MD, USA). Dulbecco’s modified Eagle medium (DMEM) and RPMI 1640 were purchased from Gibco (Grand Island, NY, USA). Fetal bovine serum (FBS) was purchased from Hyclone (Logan, UT, USA). Column chromatography was carried out on silica gel (200–300 mesh, Qingdao Marine Chemical Ltd., Qingdao, China). Thin layer chromatography (TLC) was performed on TLC silica gel 60 F254 plates. Melting points were measured using a on a Kofler hot stage apparatus and were uncorrected. ^1^H-NMR spectroscopy was performed using a Varian Unity Inova-400 spectrometer. The chemical shift values are reported in d units (ppm) relative to internal standard tetramethylsilane (TMS). ^13^C-NMR spectra were recorded on a Bruker AV II-600 MHz spectrometer. Mass spectrometry was carried out on a Waters Q-TOF Premier mass spectrometer. All solvents were dried and freshly distilled prior to use according to standard procedures. All the chemicals used were of analytical grade and commercially available. The purity of compound screened in biological assays was determined to be ≥90% by HPLC analysis with a photodiode array detector (Waters, Milford, MA, USA). An Atlantis C_18_ (150 mm × 4.6 mm, i.d. 5 μm) (Waters) was used with a gradient elution of methanol and HPLC-grade water as mobile phase at a flow rate of 1 mL/min.

### 3.2. Preparation of Compounds ***4a–j***

Compounds **4a–j** were prepared following the literature procedure [10]. 2-Chloroacetyl chloride (0.75 mol) was added dropwise to a mixture of **3a–j** (0.5 mol) and potassium carbonate (1.0 mol) in dichloromethane (550 mL) at 0 °C with stirring. After removal of the dichloromethane and vacuum filtration, the solid was washed with water and dried under vacuum for 12 h at 25–30 °C. The title compounds **4a–j** were puriﬁed by recrystallization with petroleum ether/ethyl acetate.

### 3.3. Preparation of Compounds ***6a–j***

Compounds **6a–j** were synthesized according to a literature method [11] with some modifications. Briefly, 6-aminobenzothiazole-2-thiol (35.0 mmol) was added to a mixture of **4a–j** (38.5 mmol) and triethylamine (TEA, 70.0 mmol) in tetrahydrofuran (THF, 480 mL) at room temperature with stirring for 5 h. The title compounds **6a–j** were obtained, respectively, and could be used directly for the next step without further puriﬁcation.

### 3.4. General Procedure for Preparing Compounds ***7a–t***

The reaction mixtures of compounds **6a–j** (3 mmol) were further reacted with acyl chloride (3.6 mmol) in the presence of triethylamine (TEA, 4.5 mmol) using tetrahydrofuran (THF, 30 mL) as solvent. The completion of the reaction was monitored by TLC and took 4–26 h. The precipitate was collected by filtration and washed with water to yield the crude product. Compounds **7a–t** were puriﬁed by column chromatography on silica gel using petroleum ether/ethyl acetate as eluent.

*N-(2-(2-(Benzylamino)-2-oxoethylthio)benzo[d]thiazol-6-yl)-3-chloropropanamide *(**7a**). Yellow powder, yield 72%, purity 99.8%, mp 177.2–178.1 °C; ^1^H-NMR (DMSO-*d*_6_) δ: 2.87 (t, *J* = 6.2 Hz, 2H), 3.91 (t, *J* = 6.2 Hz, 2H), 4.19 (s, 2H), 4.33 (d, *J* = 6.0 Hz, 2H), 7.22–7.28 (m, 5H), 7.55 (dd, *J* = 2.0, 4.4 Hz, 1H), 7.77 (d, *J* = 8.8 Hz, 1H), 8.40 (d, *J* = 2.0 Hz, 1H), 8.82 (t, *J* = 5.8 Hz, 1H), 10.33 (s, 1H); ^13^C-NMR (DMSO-*d*_6_) δ: 36.53, 39.49, 40.76, 42.54, 111.35, 118.49, 121.04, 126.76, 127.10, 128.19, 131.70, 135.45, 135.81, 138.80, 148.68, 148.80, 164.35, 166.43, 168.09; HRMS (ESI) *m/z*: Calcd. for C_19_H_18_ClN_3_O_2_S_2_ 420.0602; Found: 420.0592 (M-H^+^).

*N-Benzyl-2-(6-(2-bromoacetamido)benzo[d]thiazol-2-ylthio)acetamide* (**7b**). Yellow powder, yield 75%, purity 98.0%, mp 155.6–159.7 °C; ^1^H-NMR (DMSO-*d*_6_) δ: 4.08 (s, 2H), 4.20 (s, 2H), 4.33 (d, *J* = 6.0 Hz, 2H), 7.22–7.28 (m, 5H), 7.55 (d, *J* = 8.4 Hz, 1H), 7.79 (d, *J* = 8.8 Hz, 1H), 8.38 (s, 1H), 8.83 (s, 1H), 10.64 (s, 1H); ^13^C-NMR (DMSO-*d*_6_) δ: 28.72, 39.48, 42.54, 111.56, 118.65, 121.10, 126.86, 127.32, 128.29, 131.86, 135.48, 135.59, 138.94, 148.74, 148.81, 165.23, 167.36, 169.70; HRMS (ESI) *m/z*: Calcd. for C_18_H_16_BrN_3_O_2_S_2_ 449.9940; Found: 449.9929 (M-H^+^).

*2-Chloro-N-(2-(2-oxo-2-(pyridin-2-ylamino)ethylthio)benzo[d]thiazol-6-yl)acetamide* (**7c**). Yellow powder, yield 70%, purity 92.0%, mp 168.4–172.9 °C; ^1^H-NMR (DMSO-*d*_6_) δ: 4.29 (s, 2H), 4.45 (s, 2H), 7.54–7.58 (m, 2H), 7.78 (d, *J* = 8.8 Hz, 1H), 8.18–8.19 (m, 2H), 8.37 (d, *J* = 16.0 Hz, 1H), 8.89 (s, 1H), 10.35 (s, 1H), 10.54 (s, 1H); ^13^C-NMR (DMSO-*d*_6_) δ: 39.46, 43.49, 111.69, 113.46, 118.75, 119.69, 121.12, 135.42, 135.57, 138.32, 148.02, 148.91, 151.59, 164.63, 164.79, 166.20; HRMS (ESI) *m/z*: Calcd. for C_16_H_13_ClN_4_O_2_S_2_ 393.0241; Found: 393.0241 (M-H^+^).

*2-Chloro-N-(2-(2-(5-chloropyridin-2-ylamino)-2-oxoethylthio)benzo[d]thiazol-6-yl)acetamide* (**7d**). Yellow powder, yield 70%, purity 96.8%, mp 217.2–218.8 °C; ^1^H-NMR (DMSO-*d*_6_) δ: 4.29 (s, 2H), 4.44 (s, 2H), 7.50–7.54 (m, 1H), 7.77 (d, *J* = 8.8 Hz, 1H), 8.41 (s, 1H), 7.50–8.41 (m, 3H), 10.54 (s, 1H), 11.10 (s, 1H); ^13^C-NMR (DMSO-*d*_6_) δ: 39.48, 43.54, 111.80, 114.52, 118.77, 121.16, 125.38, 135.49, 135.61, 138.01, 148.79, 148.94, 150.27, 164.62, 165.37, 166.35; HRMS (ESI) *m/z*: Calcd. for C_16_H_12_Cl_2_N_4_O_2_S_2_ 426.9852; Found: 426.9822 (M-H^+^).

*N-(5-Bromopyridin-2-yl)-2-(6-(2-chloroacetamido)benzo[d]thiazol-2-ylthio)acetamide* (**7e**). Yellow powder, yield 71%, purity 96.0%, mp 221.3–222.5 °C; ^1^H-NMR (DMSO-*d*_6_) δ: 4.29 (s, 2H), 4.43 (s, 2H), 7.53 (d, *J* = 8.8 Hz, 1H), 7.76 (d, *J *= 8.8 Hz, 1H), 8.47 (s, 1H), 8.38 (s, 1H), 8.02 (m, 2H), 10.54 (s, 1H), 11.09 (s, 1H); ^13^C-NMR (DMSO-*d*_6_) δ: 39.98, 44.05, 112.29, 114.26, 115.56, 119.26, 121.66, 135.79, 135.99, 141.22, 149.12, 149.44, 151.07, 165.11, 165.23, 166.88; HRMS (ESI) *m/z*: Calcd. for C_16_H_12_BrClN_4_O_2_S_2_ 472.7864; Found: 472.7853 (M-H^+^).

*2-Chloro-N-(2-(2-(5-methylpyridin-2-ylamino)-2-oxoethylthio)benzo[d]thiazol-6-yl)acetamide* (**7f**). Yellow powder, yield 90%, purity 97.1%, mp 214.0–214.9 °C; ^1^H-NMR (DMSO-*d*_6_) δ: 2.25 (s, 3H), 4.29 (s, 2H), 4.41 (s, 2H), 7.53 (dd, *J* = 2.0, 4.4 Hz, 1H), 7.61 (dd, *J* = 2.0, 4.2 Hz, 1H), 7.77 (d, *J* = 9.2 Hz, 1H), 7.94 (d, *J* = 8.4 Hz, 1H), 8.18 (s, 1H), 8.37 (d, *J* = 1.0 Hz, 1H), 10.54 (s, 1H), 10.82 (s, 1H); ^13^C-NMR (DMSO-*d*_6_) δ: 17.22, 39.46, 43.53, 111.75, 113.13, 118.74, 121.14, 128.73, 135.30, 135.47, 139.06,147.22, 148.95, 149.20, 164.70, 164.74, 166.02; HRMS (ESI) *m/z*: Calcd. for C_17_H_15_ClN_4_O_2_S_2_ 407.0398; Found: 407.0397 (M-H^+^).

*2-Chloro-N-(2-(2-oxo-2-(pyridin-3-ylamino)ethylthio)benzo[d]thiazol-6-yl)acetamide* (**7g**). Yellow powder, yield 68%, purity 92.0%, mp 160.6–165.4 °C; ^1^H-NMR (DMSO-*d*_6_) δ: 4.29 (s, 2H), 4.45 (s, 2H), 7.55 (d, *J* = 8.0 Hz, 2H), 7.78 (d, *J* = 8.8 Hz, 1H), 8.19 (d, *J* = 8.4 Hz, 1H), 8.37 (s, 2H), 8.89 (s, 1H), 10.66 (s, 1H), 11.20 (s, 1H); ^13^C-NMR (DMSO-*d*_6_) δ: 39.43, 43.50, 111.67, 118.52, 118.72, 121.18, 130.46, 135.43, 135.51, 140.30, 148.83, 148.89, 164.36, 164.80, 166.47; HRMS (ESI) *m/z*: Calcd. for C_16_H_13_ClN_4_O_2_S_2_ 393.0241; Found: 393.0247 (M-H^+^).

*2-Chloro-N-(2-(2-(2-chloropyridin-4-ylamino)-2-oxoethylthio)benzo[d]thiazol-6-yl)acetamide* (**7h**). Yellow powder, yield 91%, purity 95.7%, mp 192.5–197.1 °C; ^1^H-NMR (DMSO-*d*_6_) δ: 4.29 (s, 2H), 4.43 (s, 2H), 7.47–7.54 (m, 2H), 7.75–7.78 (m, 2H), 8.29 (d, *J* = 5.6 Hz, 1H), 8.39 (s, 1H), 10.55 (s, 1H), 11.08 (s, 1H); ^13^C-NMR (DMSO-*d*_6_) δ: 39.48, 43.53, 111.81, 112.46, 112.62, 118.76, 121.15, 135.34, 135.49, 147.83, 148.85, 150.47, 151.02, 164.34, 164.75, 166.93; HRMS (ESI) *m/z*: Calcd. for C_16_H_12_Cl_2_N_4_O_2_S_2_ 426.9852; Found: 426.9883 (M-H^+^).

*2-Chloro-N-(2-(2-(2-chloro-4-methylpyridin-3-ylamino)-2-oxoethylthio)benzo[d]thiazol-6-yl)acetamide* (**7i**). Yellow powder, yield 80%, purity 98.0%, mp 186.4–188.3 °C; ^1^H-NMR (DMSO-*d*_6_) δ: 2.22 (s, 3H), 4.29 (s, 2H), 4.41 (s, 2H), 7.34 (d, *J* = 4.8 Hz, 1H), 7.67 (t, *J* = 8.8 Hz, 2H), 8.19 (d, *J* = 4.8 Hz, 1H), 8.55 (s, 1H), 10.24 (s, 1H), 10.37 (s, 1H); ^13^C-NMR (DMSO-*d*_6_) δ: 17.81, 39.54, 43.53, 111.87, 118.83, 121.08, 125.10, 130.49, 135.30, 135.56, 147.02, 148.67, 148.77, 148.99, 164.43, 164.76, 165.62; HRMS (ESI) *m/z*: Calcd. for C_17_H_14_Cl_2_N_4_O_2_S_2_ 441.0008; Found: 440.9992 (M-H^+^).

*2-Chloro-N-(2-(2-oxo-2-(pyrimidin-2-ylamino)ethylthio)benzo[d]thiazol-6-yl)acetamide* (**7j**). Brownish red powder, yield 63%, purity 90.0%, mp 172.6–174.9 °C; ^1^H-NMR (DMSO-*d*_6_) δ: 4.29 (s, 2H), 4.59 (s, 2H), 7.22 (t, *J* = 4.8 Hz, 1H), 7.54 (d, *J* = 4.4 Hz, 1H), 7.78 (d, *J* = 8.8 Hz, 1H), 8.38 (s, 1H), 8.69 (d, *J* = 2.4 Hz, 2H), 10.58 (s, 1H), 11.04 (s, 1H); ^13^C-NMR (DMSO-*d*_6_) δ: 39.46, 43.52, 109.98, 117.43, 121.60, 136.22, 147.29, 157.33, 157.66, 158.40, 166.22; HRMS (ESI) *m/z*: Calcd. for C_15_H_12_ClN_5_O_2_S_2_ 394.0194; Found: 394.0109 (M-H^+^).

*2-Chloro-N-(2-(2-oxo-2-(thiazol-2-ylamino)ethylthio)benzo[d]thiazol-6-yl)acetamide* (**7k**). Yellow powder, yield 78%, purity 95.0%, mp 218.3–224.7 °C; ^1^H-NMR (DMSO-*d*_6_) δ: 4.29 (s, 2H), 4.46 (s, 2H), 7.25 (d, *J* = 3.6 Hz, 1H), 7.50–7.55 (m, 2H), 7.77 (d, *J* = 8.8 Hz, 1H), 8.38 (s, 1H), 10.55 (s, 1H), 12.53 (s, 1H); ^13^C-NMR (DMSO-*d*_6_) δ: 39.46, 43.54, 111.81, 113.79, 118.77, 121.18, 135.33, 135.52, 137.71, 148.87, 157.75, 164.32, 164.74, 165.64; HRMS (ESI) *m/z*: Calcd. for C_14_H_11_ClN_4_O_2_S_3_ 398.9806; Found: 398.9816 (M-H^+^).

*2-Methoxy-N-(2-(2-oxo-2-(pyridin-2-ylamino)ethylthio)benzo[d]thiazol-6-yl)benzamide* (**7l**). Yellow powder, yield 70%, purity 92.0%, mp 154.2–158.9 °C; ^1^H-NMR (DMSO-*d*_6_) δ: 3.90 (s, 3H), 4.45 (s, 2H), 7.08 (t, *J* = 7.4 Hz, 1H), 7.14 (t, *J* = 6.0 Hz, 1H), 7.19 (d, *J* = 8.4 Hz, 1H), 7.49–7.54 (m, 1H), 7.63–7.69 (m, 2H), 7.80 (dd, *J* = 7.0, 6.4 Hz, 2H), 8.35 (d, *J* = 3.6 Hz, 1H), 8.04 (d, *J* = 8.4 Hz, 1H), 8.54 (s, 1H), 10.35 (s, 1H), 10.91 (s, 1H); ^13^C-NMR (DMSO-*d*_6_) δ: 39.46, 111.69, 118.75, 121.12, 135.41, 135.96, 148.91, 164.19, 164.57, 166.22; HRMS (ESI) *m/z*: Calcd. for C_22_H_18_N_4_O_3_S_2_ 451.0893; Found: 451.0887 (M-H^+^).

*N-(2-(2-(5-Chloropyridin-2-ylamino)-2-oxoethylthio)benzo[d]thiazol-6-yl)-2-methoxybenzamide *(**7m**). Yellow powder, yield 70%, purity 97.0%, mp 148.5–154.1 °C; ^1^H-NMR (DMSO-*d*_6_) δ: 3.90 (s, 3H), 4.45 (s, 2H), 7.19 (d, *J* = 8.4 Hz, 1H), 7.2 (t, *J* = 7.4 Hz, 2H), 7.63–7.75 (m, 2H), 7.77 (d, *J* = 8.8 Hz, 1H), 7.92 (d, *J* = 4.4 Hz, 1H), 8.08 (d, *J* = 8.4 Hz, 1H), 8.41 (d, *J* = 2.4 Hz, 1H), 8.54 (s, 1H), 10.35 (s, 1H), 11.11 (s, 1H); ^13^C-NMR (DMSO-*d*_6_) δ: 39.46, 55.84, 111.83, 111.93, 114.52, 119.17, 120.46, 120.99, 124.68, 125.38, 129.66, 132,09, 135.41, 135.95, 137.99, 146.43, 148.73, 150.28, 156.46, 164,17, 164.53, 166.37; HRMS (ESI) *m/z*: Calcd. for C_22_H_17_ClN_4_O_3_S_2_ 485.0504; Found: 485.0521 (M-H^+^).

*N-(2-(2-(5-Bromopyridin-2-ylamino)-2-oxoethylthio)benzo[d]thiazol-6-yl)-2-methoxybenzamide *(**7n**). Yellow powder, yield 75%, purity 99.0%, mp 178.4–183.5 °C; ^1^H-NMR (DMSO-*d*_6_) δ: 3.90 (s, 3H), 4.45 (s, 2H), 7.07 (t, *J* = 7.4 Hz, 1H), 7.19 (d, *J* = 8.4 Hz, 1H), 7.51 (t, *J* = 7.8 Hz, 1H), 7.66 (d, *J* = 10.8 Hz, 2H), 7.77 (d, *J* = 8.8 Hz, 1H), 7.95–8.03 (m, 2H), 8.48 (s, 1H), 8.54 (s, 1H), 10.35 (s, 1H), 11.10 (s, 1H); ^13^C-NMR (DMSO-*d*_6_) δ: 39.49, 55.87, 111.86, 111.95, 113.78, 115.08, 119.19, 120.48, 121.01, 124.69, 129.68, 132.11, 135.41, 135.96, 148.63, 148.74, 150.59, 156.48, 164.17, 164.54, 166.41; HRMS (ESI) *m/z*: Calcd. for C_22_H_17_BrN_4_O_3_S_2_ 528.9998; Found: 529.0070 (M-H^+^).

*2-Methoxy-N-(2-(2-(5-methylpyridin-2-ylamino)-2-oxoethylthio)benzo[d]thiazol-6-yl)benzamide *(**7o**). Yellow powder, yield 76%, purity 92.0%, mp 182.7–188.5 °C; ^1^H-NMR (DMSO-*d*_6_) δ: 2.28 (s, 3H), 3.90 (s, 3H), 4.45 (s, 2H), 7.08 (t, *J* = 7.6 Hz, 1H), 7.19 (d, *J* = 8.4 Hz, 1H), 7.50–7.62 (m, 2H), 7.68 (t, *J* = 10.2 Hz, 2H), 7.78 (d, *J* = 8.8 Hz, 1H), 7.95 (s, 1H), 8.18 (s, 1H), 8.52 (d, *J* = 2.0 Hz, 1H), 10.31 (s, 1H), 10.76 (s, 1H); ^13^C-NMR (DMSO-*d*_6_) δ: 17.24, 39.49, 55.86, 111.84, 111.96, 112.99, 119.18, 120.47, 121.00, 124.71, 128.61, 129.67, 132.10, 135.41, 135.95, 138.57, 147.80, 148.79, 149.49, 156.48, 164.33, 164.55, 165.90; HRMS (ESI) *m/z*: Calcd. for C_23_H_20_N_4_O_3_S_2_ 465.1050; Found: 465.1071 (M-H^+^).

*2-Methoxy-N-(2-(2-oxo-2-(pyridin-3-ylamino)ethylthio)benzo[d]thiazol-6-yl)benzamide* (**7p**). Yellow powder, yield 75%, purity 95.0%, mp 154.8–159.5 °C; ^1^H-NMR (DMSO-*d*_6_) δ: 3.90 (s, 3H), 4.45 (s, 2H), 7.08 (t, *J* = 7.6 Hz, 1H), 7.19 (d, *J* = 8.4 Hz, 1H), 7.50–7.62 (m, 2H), 7.55(d, *J* = 8.0 Hz, 1H), 7.70 (t, *J* = 6.6 Hz, 1H), 7.78 (d, *J* = 8.8 Hz, 1H), 8.29 (d, *J* = 8.4 Hz, 1H), 8.46 (d, *J* = 4.4 Hz, 1H), 8.39 (s, 1H), 8.95 (s, 1H), 10.36 (s, 1H), 11.12 (s, 1H); ^13^C-NMR (DMSO-*d*_6_) δ: 39.44, 55.62, 111.90, 112.33, 119.21, 119.95, 120.99, 121.24, 124.69, 126.23, 129.67, 132.97, 135.41, 135.96, 140.75, 144.51, 148.75, 156.47, 158.03, 164.19, 164.57, 167.32; HRMS (ESI) *m/z*: Calcd. for C_22_H_18_N_4_O_3_S_2_ 451.0893; Found: 451.0833 (M-H^+^).

*N-(2-(2-(2-Chloropyridin-4-ylamino)-2-oxoethylthio)benzo[d]thiazol-6-yl)-2-methoxybenzamide* (**7q**). Yellow powder, yield 70%, purity 90.0%, mp 177.9–180.8 °C; ^1^H-NMR (DMSO-*d*_6_) δ: 3.90 (s, 3H), 4.44 (s, 2H), 7.07 (t,* J* = 7.4 Hz, 1H), 7.19 (d,* J* = 8.0 Hz, 1H), 7.47–7.53 (m, 2H), 7.53–7.62 (m, 2H), 7.76 (t,* J* = 5.8 Hz, 1H), 7.78 (s, 1H), 8.29 (d,* J* = 5.6 Hz, 1H), 8.55 (s, 1H), 10.35 (s, 1H), 11.06 (s, 1H); ^13^C-NMR (DMSO-*d*_6_) δ: 39.44, 55.84, 111.90, 111.93, 112.47, 112.62, 119.20, 120.46, 120.99, 124.67, 129.65, 132.11, 135.41, 135.97, 148.65, 150.47, 151.03, 156.45, 163.91, 164.57, 166.95; HRMS (ESI) *m/z*: Calcd. for C_22_H_17_ClN_4_O_3_S_2_ 485.0504; Found: 485.0491 (M-H^+^).

*N-(2-(2-(2-Chloro-4-methylpyridin-3-ylamino)-2-oxoethylthio)benzo[d]thiazol-6-yl)-2-methoxybenzamide* (**7r**). Yellow powder, yield 77%, purity 90.0%, mp 166.5–168.1 °C; ^1^H-NMR (DMSO-*d*_6_) δ: 2.22 (s, 3H), 3.90 (s, 3H), 4.41 (s, 2H), 7.08 (t,* J* = 7.2 Hz, 1H), 7.19 (d, *J* = 8.4 Hz, 1H), 7.34 (d, *J* = 4.8 Hz, 1H), 7.52 (t, *J* = 7.8 Hz, 1H), 7.64 (d, *J* = 7.2 Hz, 1H), 7.69 (d, *J* = 8.8 Hz, 1H), 7.79 (d, *J* = 8.8 Hz, 1H), 8.19 (d,* J* = 4.8 Hz, 1H), 8.55 (s, 1H), 10.24 (s, 1H), 10.37 (s, 1H); ^13^C-NMR (DMSO-*d*_6_) δ: 17.84, 39.51, 55.87, 111.91, 111.97, 119.22,120.49, 120.92, 124.76, 129.65, 130.51, 132.11, 135.47, 135.96, 147.05, 148.70, 148.76, 156.48, 164.03, 164.61, 165.67; HRMS (ESI) *m/z*: Calcd. for C_23_H_19_ClN_4_O_3_S_2_ 500.0123; Found: 500.0124 (M-H^+^).

*2-Methoxy-N-(2-(2-oxo-2-(pyrimidin-2-ylamino)ethylthio)benzo[d]thiazol-6-yl)benzamide* (**7s**). Yellow powder, yield 70%, purity 90.0%, mp 169.2–171.8 °C; ^1^H-NMR (DMSO-*d*_6_) δ: 3.90 (s, 3H), 4.46 (s, 2H), 7.08 (t, *J* = 7.4 Hz, 1H), 7.18–7.24 (m, 2H), 7.52 (t, *J* = 7.6 Hz, 1H), 7.64–7.69 (m, 2H), 7.78 (d, *J* = 8.8 Hz, 1H), 8.54 (s, 1H), 8.69 (d,* J* = 8.4 Hz, 2H), 10.35 (s, 1H), 11.04 (s, 1H); ^13^C-NMR (DMSO-*d*_6_) δ: 39.46, 55.86, 111.84, 111.95, 119.17, 120.48, 121.00, 124.67, 129.68, 132.12, 135.38, 135.90, 148.80, 156.48, 157.37, 158.41, 164.45, 164.55, 166.18; HRMS (ESI) *m/z*: Calcd. for C_21_H_17_N_5_O_3_S_2_ 452.0846; Found: 452.0821 (M-H^+^).

*2-Methoxy-N-(2-(2-oxo-2-(thiazol-2-ylamino)ethylthio)benzo[d]thiazol-6-yl)benzamide* (**7t**). Yellow powder, yield 68%, purity 92.0%, mp 237.7–239.1 °C; ^1^H-NMR (DMSO-*d*_6_) δ: 3.90 (s, 3H), 4.47 (s, 2H), 7.08 (t, *J* = 7.4 Hz, 1H), 7.19 (d, *J* = 8.4 Hz, 1H), 7.25 (d, *J* = 3.6 Hz, 1H), 7.52 (t,* J* = 6.4 Hz, 2H), 7.64–7.69 (m, 2H), 7.77 (d, *J* = 8.8 Hz, 1H), 8.54 (s, 1H), 10.36 (s, 1H), 12.55 (s, 1H); ^13^C-NMR (DMSO-*d*_6_) δ: 39.37, 56.37, 112.39, 112.47, 114.32, 119.71, 120.98, 121.54, 125.24, 130.15, 132.60, 135.95, 136.50, 138.24, 149.19, 156.97, 158.27, 164.38, 165.07, 166.18; HRMS (ESI) *m/z*: Calcd. for C_20_H_16_N_4_O_3_S_3_ 457.0458; Found: 457.0457 (M-H^+^).

### 3.5. Cell Culture

Cell lines HepG2, HeLa, HCT116, SW620, SKOV-3 and MDA-MB-468 were maintained in Dulbecco’s modified Eagle medium (DMEM) containing 10% fetal bovine serum (FBS), penicillin (100 U/mL) and streptomycin (10 mg/L). Cell lines BT474, A431 and SKRB-3 were maintained in Dulbecco’s modified Eagle medium (DMEM) containing 20% fetal bovine serum (FBS), penicillin (100 U/mL) and streptomycin (10 mg/L). Cell lines H460, PC-3, A549, SW480, and BxPC-3 were maintained in RPMI 1640 containing 10% FBS, penicillin (100 U/mL) and streptomycin (10 mg/L). Cells were grown in a 5% CO_2_ incubator at 37 °C.

### 3.6. Cell Proliferation Assay (MTT Assay)

Cells (3–5 × 10^3^/well) were seeded in 200 µL of medium/well in 96-well plates (Costar Corning, Rochester, NY, USA) and cultured for 24 h. The compounds dissolved in dimethylsulfoxide (DMSO) were added to final concentration of 40 μM, 20 μM, 10 μM, 5 μM, 2.5 μM, 1.25 μM, respectively. Compounds **1** and **2** were used as positive control. After 48 h exposure, a volume of 10 μL of 10 mg/mL 3-(4,5-dimethylthiazol-2-yl)-2,5-diphenyltetrazolium bromide (MTT) was added per well and incubated for another 4 h at 37 °C, then the supernatant fluid was removed and DMSO (150 μL) was added 150 μL/well to dissolve formazan crystals for 15–20 min. The light absorptions (OD) were measured at 570 nm with SpectraMAXM5 microplate spectrophotometer (Molecular Devices). The effect of compounds on tumor cells viability was expressed by IC_50_ of each cell line. Values shown are the % viability *vs*. ctrl + SD, n = six independent experiments in triplicate.

### 3.7. Apoptosis Analysis by Flow Cytometry (FCM)

Cells were seeded into 1 mL of medium/well cell in 6-well plates (Costar Corning) culture bottles at 10 × 10^6^ cells 24 h before treatment. Then cells were treated with compound **7e** for 24 h: 0 µM (control), 0.625 µM, 1.25 µM, 2.5 µM. After 24 h incubation, floating and adherent cells were collected, washed three times with PBS (pH 7.4) and fixed for 24 h with cool alcohol at 4 °C. 1 mL cell suspension (10^6^ /mL) was washed three times with cooled PBS, treated with RNase for 30 min at 37 °C, stained it with PI for 30 min at 37 °C in a dark environment, and taken for flow cytometry analysis.

## 4. Conclusions

In conclusion, a series of novel benzothiazole-2-thiol derivatives were synthesized and their anti-proliferative activities were evaluated *in vitro*. The results showed that the pyridinyl-2-amine linked benzothiazole-2-thiol compounds **7d**, **7e**, **7f** and **7i** exhibited increased anticancer activities compared with compounds **1** and **2** on the three human cancer cells (SW480, HeLa and HepG2). Further studies showed that they displayed potent and broad-spectrum anti-proliferative activities against other human cancer cell lines. Among these compounds, compound **7e** displayed the most potent anticancer activity on SKRB-3 (IC_50_ = 1.2 nM), SW620 (IC_50_ = 4.3 nM), A549 (IC_50_ = 44 nM) and HepG2 (IC_50_ = 48 nM). The results of ﬂow cytometry analysis indicated that compound **7e** induce apoptosis in HepG2 cancer cells on a concentration-dependent manner. These data suggested that compound **7e** may be powerful tumor growth inhibitors via apoptosis as novel anticancer agents and be worth being further investigated as a potential of anticancer agent.
